# The impact of forearm immobilization and acipimox administration on muscle amino acid metabolism and insulin sensitivity in healthy, young volunteers

**DOI:** 10.1152/ajpendo.00345.2023

**Published:** 2024-01-17

**Authors:** Marlou L. Dirks, Tom S. O. Jameson, Rob C. Andrews, Mandy V. Dunlop, Doaa R. Abdelrahman, Andrew J. Murton, Benjamin T. Wall, Francis B. Stephens

**Affiliations:** ^1^Department of Public Health and Sport Sciences, Faculty of Health and Life Sciences, University of Exeter, United Kingdom; ^2^Human and Animal Physiology, Wageningen University, Wageningen, The Netherlands; ^3^Institute of Biomedical and Clinical Science, University of Exeter Medical School, Exeter, United Kingdom; ^4^National Institute for Health and Care Research Exeter Biomedical Research Centre, Exeter, United Kingdom; ^5^Department of Surgery, University of Texas Medical Branch, Galveston, Texas, United States; ^6^Sealy Center on Aging, University of Texas Medical Branch, Galveston, Texas, United States

**Keywords:** amino acid kinetics, anabolic resistance, disuse atrophy, insulin sensitivity, lipid, skeletal muscle

## Abstract

Although the mechanisms underpinning short-term muscle disuse atrophy and associated insulin resistance remain to be elucidated, perturbed lipid metabolism might be involved. Our aim was to determine the impact of acipimox administration [i.e., pharmacologically lowering circulating nonesterified fatty acid (NEFA) availability] on muscle amino acid metabolism and insulin sensitivity during short-term disuse. Eighteen healthy individuals (age: 22 ± 1 years; body mass index: 24.0 ± 0.6 kg·m^−2^) underwent 2 days forearm immobilization with placebo (PLA; *n* = 9) or acipimox (ACI; 250 mg Olbetam; *n* = 9) ingestion four times daily. Before and after immobilization, whole body glucose disposal rate (GDR), forearm glucose uptake (FGU; i.e., muscle insulin sensitivity), and amino acid kinetics were measured under fasting and hyperinsulinemic-hyperaminoacidemic-euglycemic clamp conditions using forearm balance and l-[*ring*-^2^H_5_]-phenylalanine infusions. Immobilization did not affect GDR but decreased insulin-stimulated FGU in both groups, more so in ACI (from 53 ± 8 to 12 ± 5 µmol·min^−1^) than PLA (from 52 ± 8 to 38 ± 13 µmol·min^−1^; *P* < 0.05). In ACI only, and in contrast to our hypothesis, fasting arterialized NEFA concentrations were elevated to 1.3 ± 0.1 mmol·L^−1^ postimmobilization (*P* < 0.05), and fasting forearm NEFA balance increased approximately fourfold (*P* = 0.10). Forearm phenylalanine net balance decreased following immobilization (*P* < 0.10), driven by an increased rate of appearance [from 32 ± 5 (fasting) and 21 ± 4 (clamp) preimmobilization to 53 ± 8 and 31 ± 4 postimmobilization; *P* < 0.05] while the rate of disappearance was unaffected by disuse or acipimox. Disuse-induced insulin resistance is accompanied by early signs of negative net muscle amino acid balance, which is driven by accelerated muscle amino acid efflux. Acutely elevated NEFA availability worsened muscle insulin resistance without affecting amino acid kinetics, suggesting increased muscle NEFA uptake may contribute to inactivity-induced insulin resistance but does not cause anabolic resistance.

**NEW & NOTEWORTHY** We demonstrate that 2 days of forearm cast immobilization in healthy young volunteers leads to the rapid development of insulin resistance, which is accompanied by accelerated muscle amino acid efflux in the absence of impaired muscle amino acid uptake. Acutely elevated fasting nonesterified fatty acid (NEFA) availability as a result of acipimox supplementation worsened muscle insulin resistance without affecting amino acid kinetics, suggesting increased muscle NEFA uptake may contribute to inactivity-induced insulin resistance but does not cause anabolic resistance.

## Graphical abstract

**Figure:**
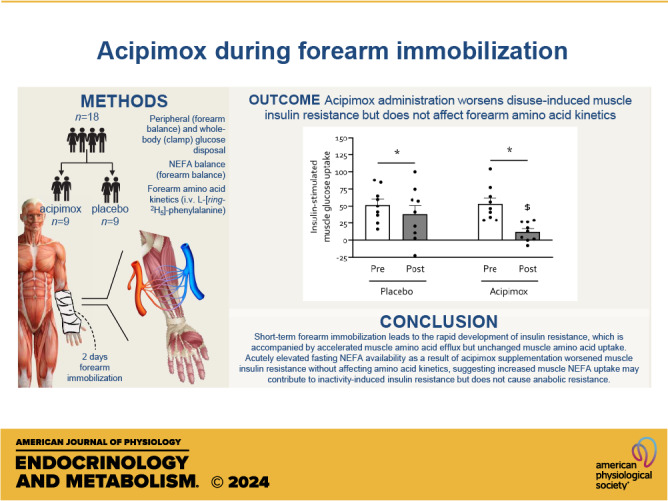

## INTRODUCTION

Short periods of muscle disuse, e.g., during illness or recovery from injury, lead to rapid and substantial muscle atrophy, which is associated with negative consequences including a loss of muscle strength and function (1–4). This loss of muscle mass is caused by negative net muscle protein balance, likely largely driven by impaired muscle protein synthesis in the fasting and postprandial states, the latter termed anabolic resistance (5). We have recently shown that postprandial muscle amino acid uptake is reduced following 7 days of immobilization (6), suggesting that anabolic resistance might (partially) be caused by limited intramuscular amino acid availability following protein ingestion. In parallel with changes in muscle amino acid metabolism, disuse also leads to the development of muscle insulin resistance, i.e., a 30–40% reduction in insulin-stimulated skeletal muscle glucose uptake (1, 7–9), which we have previously demonstrated to be maximally developed within 2 days of removing muscle contraction (10, 11). Disuse-induced muscle anabolic and insulin resistance are clearly due to a lack of contractile stimuli which otherwise maintain or increase muscle amino acid and glucose metabolism, but the underlying metabolic mechanisms are yet to be elucidated.

Perturbations in muscle lipid handling have been suggested to underpin the development of anabolic and insulin resistance during muscle disuse. We have previously demonstrated that a shift toward positive nonesterified fatty acid (NEFA) balance occurs across the forearm in response to ingestion of a mixed meal after 2 and 7 days of immobilization, which corresponded with insulin (10) and anabolic (5, 6, 12) resistance. Presumably this positive balance results in lipid accumulation within the muscle during disuse. Indeed, changes in intramuscular diacylglycerol metabolism occur in the first 7 days of disuse (1, 13), and more prolonged disuse (>7 days) is associated with intramyocellular lipid accumulation (14), implicating altered muscle lipid handling as a locus of control for insulin and anabolic resistance during disuse. In support, we have previously shown that increasing plasma NEFA concentrations by experimental intravenous lipid infusion directly induces both insulin and anabolic resistance (15) and that a high-fat hypercaloric diet during 7 days of immobilization exacerbates the disuse-induced blunting of postprandial forearm amino acid balance (6). Thus this raises the question of whether preventing the immobilization-induced increase in forearm NEFA balance can reduce anabolic and insulin resistance and, ultimately (partially), ameliorate the muscle deterioration associated with disuse.

To address this hypothesis, we performed a double-blind, randomized controlled study to investigate the impact of pharmacologically suppressing circulating NEFA availability and, therefore, muscle lipid accumulation during 2 days of forearm immobilization on muscle amino acid metabolism and whole body and muscle insulin sensitivity for the first time. We used four times daily administration of 250 mg acipimox, a nicotinic acid analog that inhibits adipose tissue lipolysis for around 6 h and improves insulin sensitivity (16–18) so that muscle NEFA uptake would be reduced throughout the entire immobilization period. Measurements of muscle glucose, amino acid, and NEFA balance were performed using the arteriovenous-deep venous forearm balance technique (6, 10) in the fasting state and during a hyperinsulinemic-hyperaminoacidemic-euglycemic clamp. This permitted us to directly measure insulin sensitivity in a controlled “postprandial” steady-state, before and immediately after 2 days of forearm immobilization. To provide further insight into physiological mechanisms underlying any changes in anabolic sensitivity, intravenous l-[*ring*-^2^H_5_]-phenylalanine infusions were used in parallel to measure rates of forearm amino acid disappearance (*R*_d_) and appearance (*R*_a_).

## METHODS

### Participants

Twenty-two healthy, young males and females were included in the present study. The participant characteristics of the final 18 participants (please see results for details on dropouts) included in the study are depicted in Table 1. Before inclusion in the study, participants attended the Clinical Research Facility (CRF) at the Royal Devon University Healthcare NHS Foundation Trust for a routine medical screening to ensure their eligibility to take part. Participants were excluded if they fulfilled one or more of the following criteria: age below 18 or over 40 years, body mass index below 18.5 or over 30 kg·m^−2^, metabolic impairment (e.g., type 1 or 2 diabetes), hypertension, cardiovascular disease, chronic use of any prescribed over the counter pharmaceuticals or nutritional supplements, a personal or family history of thrombosis/epilepsy/seizures/schizophrenia, known allergies for any of the pharmacological treatments, any disorders in muscle or lipid metabolism, presence of an ulcer in the stomach or gut, severe kidney problems, and pregnancy. All participants were informed of the nature and risks of the experiment before oral, and written informed consent was obtained. Height and weight were measured, and body composition was determined by air displacement plethysmography (Bodpod; Life Measurement, Inc., Concord, CA). The present study was approved by the National Health Service (NHS) Wales REC4 Research Ethics Committee in accordance with the Declaration of Helsinki (version October 2013). This study was part of a larger trial investigating the effects of pharmacological manipulations of substrate availability on muscle health during forearm immobilization, registered on clinicaltrials.gov as NCT03866512.

**Table 1.** Participant characteristics

	PLA (*n* = 9)	ACI (*n* = 9)
Sex, males/females	5/4	4/5
Age , yr	23 ± 2	20 ± 1
Height , cm	175 ± 3	172 ± 2
Body mass kg	72.9 ± 3.1	68.6 ± 3.2
BMI, kg·m^−2^	23.9 ± 0.7	22.6 ± 0.9
Body fat, %body mass	25.1 ± 3.1	21.9 ± 3.1
Lean mass, kg	54.8 ± 3.8	53.5 ± 2.1
Systolic blood pressure, mmHg	114 ± 4	116 ± 4
Diastolic blood pressure, mmHg	65 ± 2	70 ± 3

Data are expressed as means ± SE. PLA, placebo; ACI, acipimox. All variables *P* > 0.05.

### Experimental Overview

Following inclusion, participants visited the CRF for a baseline metabolic test day during which fasting and postprandial forearm glucose uptake (FGU), and amino acid kinetics were measured using the arterialized venous-deep venous (AV-V) forearm balance method. Participants attended the CRF for the application of a forearm cast (i.e., to immobilize the wrist), which signified the beginning of the 2-day immobilization period. During these 48 h, participants were randomized into receiving one of the following two pharmacological treatments in a double-blind manner: 250 mg acipimox (ACI; to pharmacologically lower circulating NEFA availability and thereby attenuate muscle lipid accumulation, during immobilization) or placebo (PLA), all to be taken four times daily. During those same 2 days, participants were provided with a fully controlled eucaloric diet. Following 2 days of forearm immobilization, pharmacological treatment, and standardized nutrition, the metabolic test day was repeated. The forearm cast was removed following the final test day.

### Metabolic Test Day

At 0800, after an overnight fast from 2200, participants arrived at the CRF for the metabolic test day. For females not using hormonal/intrauterine contraceptives, both test days were scheduled on one of the first 10 days of their menstrual cycle, i.e., the follicular phase. For females on oral contraceptives both test days were conducted outside the stop week. Participants rested on the bed in a semi-supine position for the entire metabolic test day. Intravenous cannulas were placed *1*) anterograde in an antecubital vein of the nonimmobilized hand for intravenous infusions, *2*) retrograde into a dorsal hand vein of the nonimmobilized hand for arterialized venous blood sampling, and *3*) retrograde into a deep-lying antecubital vein of the (to-be) immobilized arm to sample venous blood draining the forearm muscle bed (19, 20). The cannulated hand (*cannula 2*) was placed in a heated (55°C) hand warmer. Following collection of a baseline venous blood sample, a primed (0.5 mg·kg body weight^−1^), continuous (0.5 mg·kg body weight^−1^·h^−1^) infusion of l-[*ring*-^2^H_5_]phenylalanine (CK Isotopes Ltd., Newtown Unthank, UK) was started for the duration of the test day (*t* = −150 min). Arterialized-venous (AV) and deep-venous (V) blood was sampled simultaneously five times between *t* = −30 and *t* = 0 min to measure fasting forearm muscle metabolism. Brachial artery blood flow of the (to-be) immobilized arm was determined by high-resolution ultrasound imaging in duplex mode (∼12 MHz, Apogee 1000, SIUI) before every blood sample. Luminal diameter was imaged 5 cm proximal to the antecubital fossa for a 2-s period. At the same anatomic location mean blood velocity was determined by integration of the pulsed-wave Doppler signal for a minimum of 8 cardiac cycles (21). Semiautomatic analyses of captured files were done using Brachial Analyzer for Research, version 6.10.2 (Medical Imaging Applications LLC, Coralville, IA; Ref. 22).

At *t* = 0 min, a hyperinsulinemic-hyperaminoacidemic-euglycemic clamp was started to examine postprandial forearm muscle metabolism. Hyperinsulinemic-euglycemic clamps allow for repeated steady-state forearm balance measurements allowing to detection of a surplus effect of a potential intervention on top of the already large impact of immobilization (i.e., ∼40% decrease in both muscle glucose uptake and muscle protein synthesis following 7 days of limb immobilization; Refs. 10, 23), but are also regarded as the gold-standard technique to measure whole body glucose disposal. This allows interpretation of effects on the local forearm level in the light of potential changes in whole body glucose disposal, currently not possible when these techniques are used in isolation. Since hyperinsulinemic-euglycemic clamps lead to a suppression of circulating amino acids due to insulin-induced suppression of protein breakdown (31, 32), the use of intravenous amino acid co-infusion induces a steady state situation with postprandial amino acid concentrations. Therefore, the following intravenous infusions were started in the antecubital elbow vein: a primed (0–5 min: 128.2 mU·m^2^·min^−1^; 5–10 min: 71.8 mU·m^2^·min^−1^), continuous (from 10 min: 50 mU·m^2^·min^−1^) infusion of insulin (Actrapid, Novo Nordisk Ltd, Gatwick, UK), and a primed (0.46 mL·kg body weight^−1^) continuous (1.38 mL·kg body weight^−1^·h^−1^) infusion of 10% Primene (Baxter Healthcare Ltd, Northampton, UK) which was spiked with 7% l-[*ring*-^2^H_5_]phenylalanine to minimize plasma tracer dilution. A variable rate of 20% dextrose (Baxter) infusion was started in the same cannula. Every 5 min throughout the entire 3-h clamp a 0.5-mL blood sample was taken to determine blood glucose concentration, and the amount of glucose infused was altered to maintain euglycemia at 5.0 mmol·L^−1^. Potassium chloride (0.3% KCl in 0.9% NaCl; Baxter) was infused in the (to-be) immobilized arm at a rate of 1 mL·kg body weight^−1^·h^−1^ to prevent insulin-induced hypokalemia. The first 12 participants completed the study without issues. Thereafter, unexplainable nausea and sickness occurred at the end of the clamp in two participants (of which one dropped out). The final four volunteers in the study received prophylactic metoclopramide hydrochloride (10 mg) intravenously at *t* = 120 min to prevent these issues. Metoclopramide infusion did not affect any of the observed results. Every 30 min from the start of the clamp, brachial artery blood flow was measured and AV and V blood was sampled simultaneously (by 2 different investigators). During the last half hour of the clamp (i.e., between *t* = 150 and *t* = 180 min), five simultaneous AV and V blood samples were collected to measure insulin-stimulated forearm muscle metabolism. The same steady-state period was used to calculate the mean glucose disposal rate (GDR).

Forearm glucose uptake and forearm nonesterified fatty acid (NEFA) balance were calculated as the AV-V difference in glucose and NEFA concentrations, respectively, multiplied by brachial artery blood flow (24), as reported previously (10). Forearm amino acid kinetics were calculated as described previously (6). As forearm volume correlated well with body weight in our previous work (6) (Pearson’s correlation 0.779, *P* < 0.001) and did not change with 7 days of forearm immobilization (6), in the present study we estimated forearm volume by multiplying body weight by 12.7 to use in the calculations for amino acid kinetics.

### Forearm Immobilization

On the morning of the start of the 2-day forearm immobilization period, participants arrived at the CRF at 0800 to have a forearm cast fitted. Firstly, stockinette and undercast padding were applied to protect the skin. Next, a fiberglass (Benecast, BeneCare Medical, Manchester, UK) cast was fitted to the forearm and hand to immobilize the wrist. This resulted in a cast that extended from 5 cm distal of the antecubital fossa to 2 cm proximal of the fingertips, which restricted wrist flexion, extension, abduction, adduction, supination, and pronation. Participants were provided with a sling and instructed to wear that during all waking hours to keep the hand elevated above the elbow. A waterproof cover was provided to keep the cast dry while showering. The immobilized arm was randomized and counterbalanced for arm dominance. Body weight was measured after application of the cast and this was repeated at the start of the second metabolic test day.

### Pharmacological Treatment

During the 2 days of forearm immobilization, participants were randomly allocated to receive one of the following two pharmacological treatments in a double-blind manner: 250 mg acipimox (Olbetam, Pfizer Ltd, Sandwich, UK), or an inert placebo (containing microcrystalline cellulose, lactose, and magnesium stearate, manufactured by the Guy’s and St Thomas’ NHS Foundation Trust Pharmacy Manufacturing Unit). Treatments were prepared by the Royal Devon University Healthcare NHS Foundation Trust Clinical Trials Pharmacy and dispensed in opaque containers by a CRF research nurse blinded to treatment. Both treatments were orally ingested four times daily, i.e., at 0800, 1300, 1800, and 2300 (with the final dose on the second day taken at 2200). Participants were instructed to take their treatment with water, and with/immediately after a meal or snack. Compliance was monitored via provided treatment logs, returned containers, and daily communication with study participants.

### Dietary Intake

Before the immobilization period participants were instructed to keep a food diary for 3 consecutive days, including 2 weekdays and 1 weekend day. These food diaries were used to calculate habitual energy and macronutrient intake using the online licensed Nutritics software (25). During the 2 days of forearm immobilization, participants received a fully-controlled eucaloric diet as described previously (10). All meals and snacks were provided, whereas water and noncaloric drinks were allowed ad libitum. Energy requirements were individually calculated as basal metabolic rate (BMR via Henry Equations; Ref. 26) multiplied by an activity factor (International Physical Activity Questionnaire; Ref. 27). The diet was designed to provide 1.2 g protein·kg body weight^−1^·day^−1^, with a target macronutrient composition of 50–55 energy percent (en%) carbohydrate, 30–35 en% fat, 10–15 en% protein, and 2 en% dietary fiber. Compliance with the provided diet was assessed via completed 2-day food diaries, returned food containers, and daily communication with study participants.

### Sample Analyses

Arterialized venous and deep-venous blood samples were collected for determination of whole blood glucose, plasma amino acid concentrations, and stable isotope enrichments, and serum insulin and NEFA concentrations. Therefore, one part of every sample (1 mL) was collected in a BD Vacutainer fluoride/oxalate tube, rolled on a tube roller for 2 min to inhibit glycolysis, and subsequently analyzed for whole blood glucose concentrations (YSI 2500 blood glucose analyzer, Xylem Analytics UK, Tunbridge Wells, UK). A second part (5 mL) was collected in BD Vacutainer SST II tubes, which were left to clot at room temperature for ≥30 min and then centrifuged at 2,500 *g* at 4°C for 10 min to obtain serum samples. Arterialized serum samples were used to determine insulin concentrations (human insulin ELISA kit, DX-EIA-2935; Oxford Biosystems Ltd, Milton Park, UK). Serum NEFA concentrations were measured spectrophotometrically in arterialized venous and deep-venous serum samples (FA115 kit, Randox Laboratories Ltd, Crumlin, UK). A third part of every sample (4 mL) was collected in BD Vacutainer PST Lithium Heparin tubes and immediately centrifuged at 2,500 *g* at 4°C for 10 min to obtain plasma samples. Plasma amino acid concentrations and l-[*ring*-^2^H_5_]phenylalanine enrichments were analyzed using gas chromatography-mass spectrometry as described previously (6).

### Statistics

All data are expressed as means ± SE Baseline characteristics between groups were tested using an independent samples *t*-test. Data were analyzed using a repeated measures ANOVA with immobilization (pre vs. post), prandial state (fasting vs. clamp), and/or time point (during test day) as within-subjects factors, and treatment (ACI vs. PLA) as between-subjects factor. In case of a significant interaction additional repeated measures ANOVAs were performed, with subsequent Bonferroni post hoc tests applied where necessary to locate individual differences. Statistical data analysis was performed using SPSS version 27.0 (IBM Corp, Armonk, NY). Statistical significance was set at *P* < 0.05.

## RESULTS

### Participants and Dietary Intake

The two treatment groups did not differ in any baseline characteristics (Table 1) or habitual dietary intake (Table 2) before the start of the study. Three participants dropped out during the study: two because of cannulation issues on the first metabolic test day, and one because of issues with nausea and sickness. One participant in the acipimox group was excluded as both their whole body glucose disposal and forearm glucose uptake at baseline were >2 SD greater than the rest of the population, despite being classified as recreationally active. The standardized diet consumed during forearm immobilization contained more energy than their habitual diet (*P* < 0.05) due to absolute and relative increases in dietary carbohydrate and fiber content (both *P* < 0.05), whereas alcohol intake was removed. Specifically, fiber en% in the habitual and immobilization diets was 1.94 ± 0.19 and 2.28 ± 0.14 (PLA) and 2.08 ± 0.26 and 2.17 ± 0.09 (ACI), respectively (*P* < 0.05 for effect of controlled diet, *P* > 0.05 for interaction and treatment effects). Although relative protein content of the diet decreased when compared with habitual intake (*P* < 0.05), absolute protein intake was unchanged due to an increase in energy intake. No differences were observed in dietary intake between groups (all *P* > 0.05). During the 2 days of forearm immobilization body weight decreased from 73.8 ± 3.0 to 73.4 ± 3.1 kg in PLA and from 69.8 ± 2.9 to 69.0 ± 3.0 kg in ACI (*P* < 0.05), with no differences between groups (*P* > 0.05).

**Table 2.** Dietary intake

	PLA (*n* = 9)		ACI (*n* = 9)	
	Habitual	Immobilization	Habitual	Immobilization
Energy, MJ·day^−1^	9.4 ± 0.9	11.6 ± 0.6*	8.6 ± 1.0	11.0 ± 0.5*
Protein, g·kg^−1^·day^−1^	1.19 ± 0.12	1.22 ± 0.02	1.08 ± 0.09	1.22 ± 0.01
Protein, g·day^−1^	88 ± 9	91 ± 4	74 ± 8	84 ± 4
Carbohydrates, g·day^−1^	224 ± 18	371 ± 24*	235 ± 30	349 ± 15*
Fat, g·day^−1^	86 ± 14	96 ± 4	79 ± 11	92 ± 5
Fibers, g·day^−1^	21 ± 3	32 ± 2*	20 ± 2	28 ± 1*
Alcohol, g·day^−1^	18 ± 12	0 ± 0*	14 ± 9	0 ± 0*
Protein, en%	17 ± 1	13 ± 0*	15 ± 1	13 ± 0*
Carbohydrate, en%	42 ± 2	53 ± 1*	45 ± 1	54 ± 0*
Fat, en%	35 ± 3	31 ± 1	34 ± 2	31 ± 0
Fibers, en%	2 ± 0	2 ± 0*	2 ± 0	2 ± 0*
Alcohol, en%	5 ± 3	0 ± 0*	4 ± 2	0 ± 0*

Data are expressed as means ± SE. PLA, placebo; ACI, acipimox; en%, energy percentage. No differences were observed in habitual dietary intake between groups (all variables *P* > 0.05). *Significantly different from corresponding habitual intake value (*P* < 0.05).

### Nonesterified Fatty Acids

**Figure 1.**
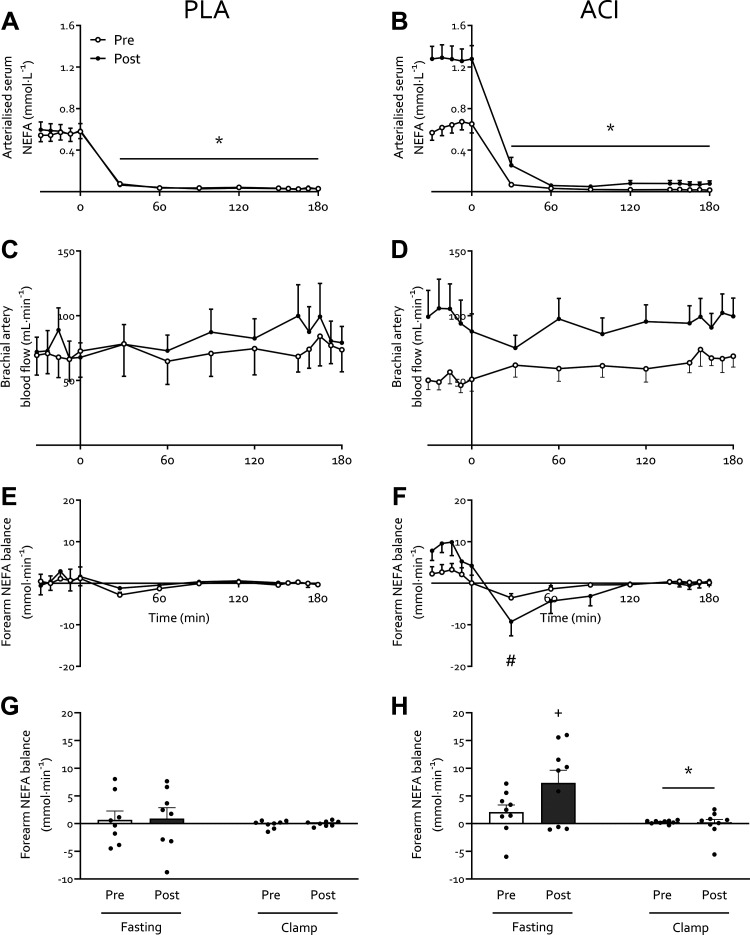
Nonesterified fatty acid (NEFA) concentrations and balance before and immediately following 2 days of forearm immobilization in healthy volunteers supplemented with placebo (PLA; *n* = 8; *A*, *C*, *E*, and *G*) or acipimox (ACI; *n* = 9; *B*, *D*, *F*, and *H*) 4 times daily. *A* and *B*: arterialized NEFA concentrations in the fasting state and during the 3-hour hyperinsulinemic-hyperaminoacidemic-euglycemic clamp. *C* and *D*: brachial artery blood flow, measured via Doppler ultrasound, which is used to calculate forearm NEFA balance. *E* and *F*: forearm NEFA balance over time, with positive and negative values indicating a net uptake and release of NEFA in forearm tissues, respectively. *G* and *H*: average NEFA balance in the fasting state and during the clamp. Data are expressed as means ± SE. *Significantly different from fasting (*P* < 0.05). #Significantly different from *t* = −22.5 min (*P* < 0.05). +Trend for difference from preimmobilization value (*P* < 0.10).

No differences in fasting serum NEFA concentrations were observed between groups before the study (*P* > 0.05). Fasting arterialized serum NEFA concentrations increased with immobilization in both groups (immobilization effect *P* > 0.05; Fig. 1, *A* and *B*), an effect which was driven by an increase in ACI (from 0.63 ± 0.08 to 1.28 ± 0.12 mmol·L^−1^; *P* < 0.05) but not PLA (from 0.56 ± 0.06 to 0.58 ± 0.07 mmol·L^−1^; *P* = 0.591). For arterialized serum NEFA concentrations during the clamp, all main effects and interactions were statistically significant (all *P* < 0.05). In both groups, hyperinsulinemic-hyperaminoacidemic-euglycemia suppressed arterialized NEFA concentrations (*P* < 0.05). The significant interaction effects were attributed to fasting NEFA concentrations being elevated following immobilization in ACI but not in PLA.

Brachial artery blood flow (Fig. 1, *C* and *D*) increased with immobilization (*P* < 0.05) but to a greater extent in ACI (interaction *P* < 0.10) and particularly in the fasted state (*P* < 0.05). Fasting forearm NEFA balance tended to increase with immobilization in ACI (*P* = 0.10) but not in PLA (*P* = 0.829; Fig. 1, *E* and *F*). Forearm NEFA balance demonstrated a time effect and time × treatment interaction (both *P* < 0.05), which were attributed to a time effect and immobilization × time interaction (both *P* < 0.05) in ACI only. Specifically, this was due to a lower forearm NEFA balance at 30 min following the start of the clamp when compared to the *t* = −22.5 min fasting value (Fig. 1*F*). As a result, the average forearm NEFA balance was reduced during the clamp when compared to the fasting state (*P* < 0.05), with no effect of immobilization (*P* > 0.05) but a trend for overall higher values in ACI (*P* < 0.10) and for a clamp × treatment interaction (*P* < 0.10; Fig. 1*H*).

### Whole Body Insulin Sensitivity

**Figure 2.**
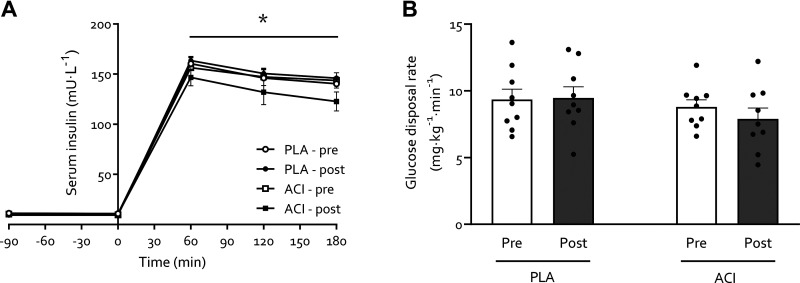
*A*: serum insulin concentrations during a 3-hour 50 mU·m^2^·min^−1^ hyperinsulinemic-hyperaminoacidemic-euglycemic clamp in young healthy volunteers undergoing 2 days of forearm immobilization with placebo (PLA; *n* = 9) or acipimox (ACI; *n* = 9) supplementation. *B*: glucose disposal rates during the final 30 min of the 3-hour clamp, representing steady-state conditions. Data was analyzed using repeated measures ANOVAs. Data are expressed as means ± SE. *Significantly different from fasting concentrations (*P* < 0.05).

No differences were found in fasting blood glucose or serum insulin concentration, and glucose disposal rate (GDR), between PLA and ACI during the preimmobilization test day (all *P* > 0.05). Fasting blood glucose concentration decreased in both groups with immobilization (*P* < 0.05) but to a greater extent in ACI (interaction: *P* < 0.05), i.e., from 4.51 ± 0.14 to 4.45 ± 0.07 in PLA and from 4.42 ± 0.12 to 3.95 ± 0.10 mmol·L^−1^ in ACI. Fasting serum insulin concentration (Fig. 2*A*) remained unchanged during forearm immobilization in both groups (*P* > 0.05), with values being 11.0 ± 0.8 and 9.4 ± 1.0 mU·L^−1^ in PLA and 10.6 ± 1.0 and 11.0 ± 1.2 mU·L^−1^ in ACI on the pre- and postimmobilization test days, respectively. During both pre- and postimmobilization clamps, circulating serum insulin concentration peaked at 160 ± 6 mU·L^−1^ at *t* = 60 min and averaged 145 ± 5 mU·L^−1^ at the end of the clamps, with no differences between groups (*P* > 0.05). GDR displayed no significant effects or interaction (Fig. 2*B*; all *P* > 0.05).

### Muscle Insulin Sensitivity

**Figure 3.**
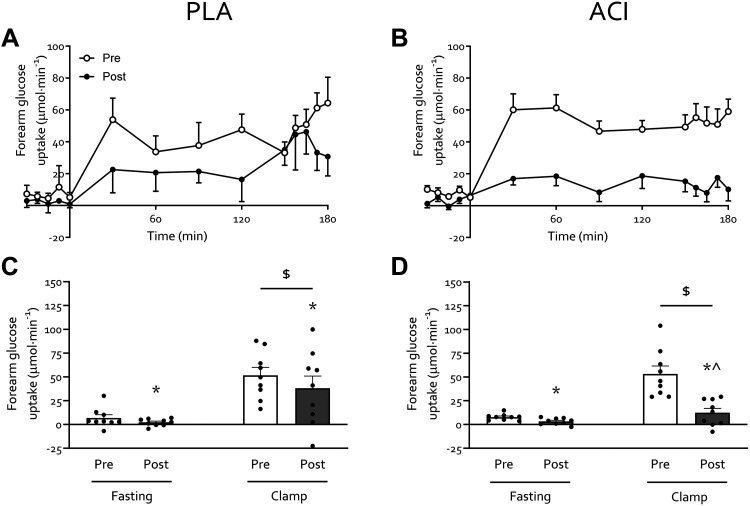
Muscle glucose uptake following 2 days of forearm immobilization with placebo (PLA; *n* = 9; *A* and *C*) or acipimox (ACI; *n* = 9; *B* and *D*) supplementation in healthy young volunteers. *C* and *D*: average forearm glucose uptake (calculated using brachial artery blood flow; Fig. 1, *C* and *D*) in the fasting state and during the clamp. Data are expressed as means ± SE. *Significantly different from preimmobilization. $Significantly different from fasting (*P* < 0.05). ^Significantly different from preimmobilization clamp value (*P* < 0.05).

Fasting forearm glucose uptake (FGU; Fig. 3, *A*–*D*) was not different between treatments on the preimmobilization test day (*P* > 0.05). FGU increased on average threefold from fasting during the hyperinsulinemic-hyperaminoacidemic-euglycemic clamp on both pre- and postimmobilization test days (*P* < 0.05). Two days of forearm immobilization led to a reduction in both fasting and insulin-stimulated FGU (*P* < 0.05). Based on a trend for an immobilization × treatment interaction (*P* = 0.097) groups were analyzed separately and demonstrated an immobilization × clamp interaction (*P* < 0.05) in ACI only. This implies participants in ACI were unable to increase FGU during the postimmobilization clamp when compared to the fasting state (*P* > 0.05), whereas the insulin-stimulated state still led to increased FGU in PLA (*P* < 0.05). In other words, ACI led to impaired insulin-stimulated FGU following 2 days of forearm immobilization, an effect confirmed by a significant difference between pre- and postimmobilization insulin-stimulated FGU (paired *t*-test, ACI: *P* < 0.05, PLA: *P* > 0.05). These findings occurred despite increased brachial artery blood flow on the postimmobilization test day in ACI only (*P* < 0.05; Fig. 1*D*).

### Amino Acid Concentrations and Kinetics

**Figure 4.**
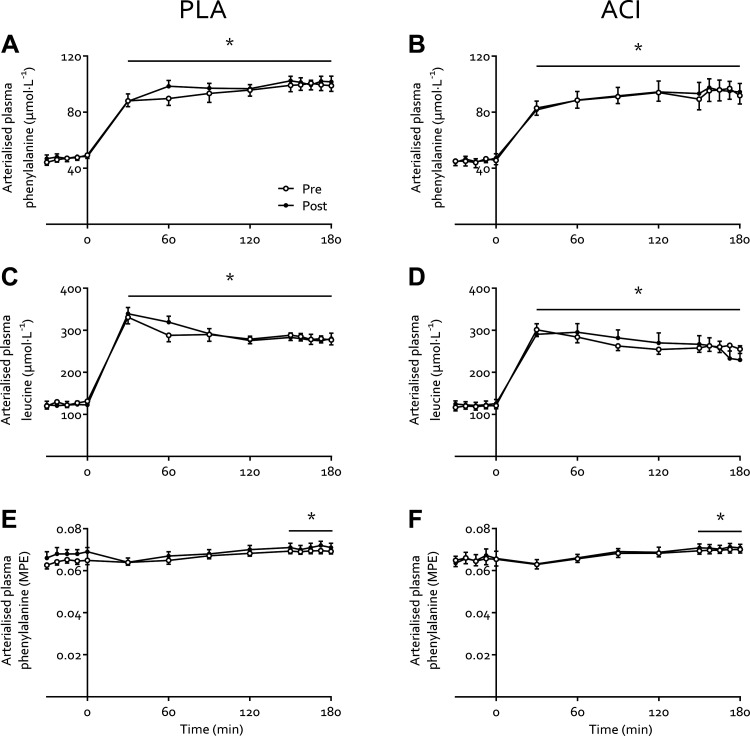
Arterialized plasma leucine concentrations (*A* and *B*), phenylalanine concentrations (*C* and *D*), and l-[*ring*-^2^H_5_]phenylalanine enrichments (*E* and *F*) before and immediately following 2 days of forearm immobilization in healthy young volunteers, in the fasting state −30 to 0 min), and during a 3-hour hyperinsulinemic-hyperaminoacidemic-euglycemic clamp (0 to 180 min). Participants were supplemented with placebo (PLA; *n* = 9; *A*, *C*, and *E*) or acipimox (ACI; *n* = 8; *B*, *D*, and *F*) while consuming a fully controlled diet. Data are expressed as means ± SE. *Significantly higher than fasting values (*P* < 0.05).

Arterialized venous plasma leucine and phenylalanine concentrations increased from 125 ± 4 and 47 ± 1 to 272 ± 7 and 98 ± 3 µmol·L^−1^, respectively, during the transition from the fasting state to hyperinsulinemic-hyperaminoacidemic-euglycemic clamp conditions (*P* < 0.05) and were not affected by immobilization or treatment (both *P* > 0.05; Fig. 4). Plasma ^2^H_5_-phenylalanine enrichments (Fig. 4, *E* and *F*) increased moderately during the clamp (from 0.066 ± 0.001 to 0.070 ± 0.001 mole percent excess (MPE) in the fasting state and during clamp, respectively; *P* < 0.05) but were not affected by immobilization or treatment (both *P* > 0.05).

**Figure 5.**
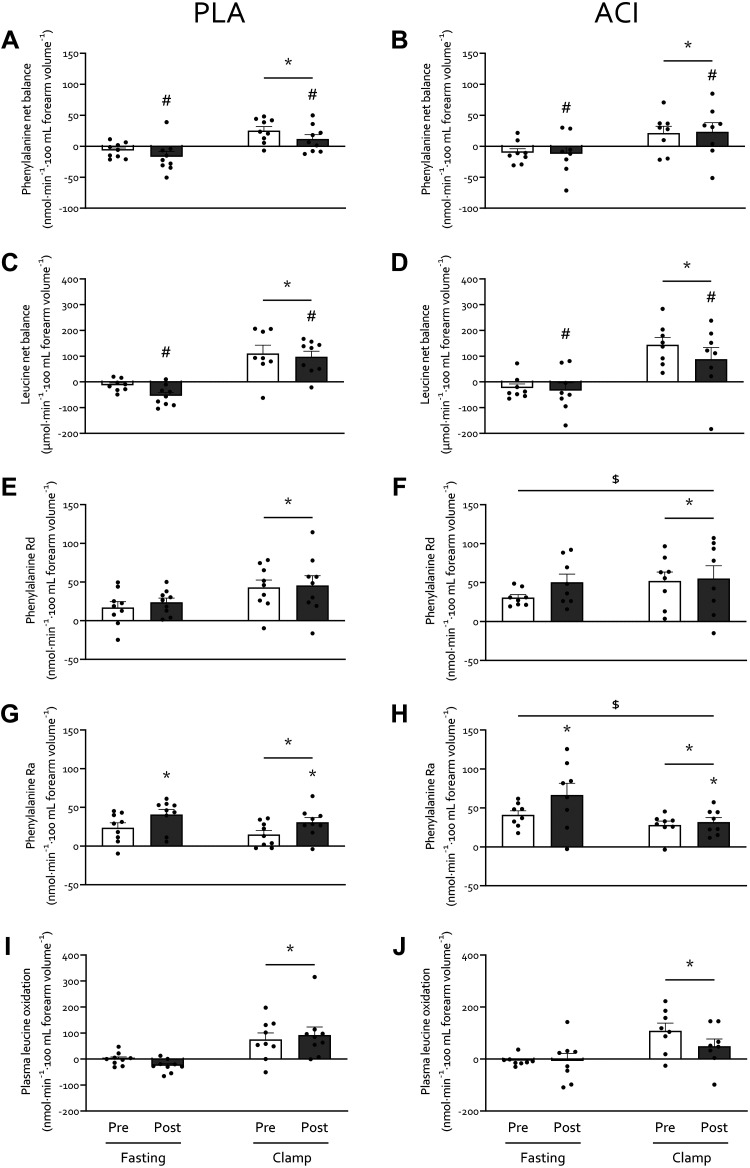
Amino acid kinetics before (white bars) and immediately after (gray bars) 2 days of forearm immobilization in healthy volunteers supplemented with placebo (*n* = 9; *A*, *C*, *E*, *G*, and *I*) or acipimox (*n* = 8; *B*, *D*, *F*, *H*, and *J*), in the fasting state and during the final 30 min of a 3-hour hyperinsulinemic-hyperaminoacidemic-euglycemic clamp. *A*−*D*: leucine (*A* and *B*) and phenylalanine (*C* and *D*) net balance. *E*−*H*: phenylalanine rate of disappearance (*R*_d_; *E* and *F*; i.e., measure of muscle amino acid uptake) and rate of appearance (*R*_a_; *G* and *H*; measure of muscle protein breakdown). *I* and *J*: plasma leucine oxidation rates. PLA, placebo; ACI, acipimox. Data are expressed as means ± SE. *Significantly different from fasting (*P* < 0.05). *#Effect of immobilization (*P < 0.05; #*P* < 0.10). $Significantly higher than PLA group (*P* < 0.05).

Forearm net balance (NB) of both phenylalanine (Fig. 5, *A* and *B*) and leucine (Fig. 5, *C* and *D*) switched from negative (−13 ± 4 and −32 ± 9 nmol·min^−1^·100 mL forearm volume^−1^, respectively) to positive (17 ± 6 and 109 ± 16 nmol·min^−1^·100 mL forearm volume^−1^, respectively; *P* < 0.05) from fasting to clamp conditions. Immobilization decreased leucine forearm NB (*P* < 0.05) and tended to decrease phenylalanine forearm NB (*P* < 0.10), with no effect of treatment (*P* > 0.05). Forearm phenylalanine rate of disappearance (*R*_d_; Fig. 5, *E* and *F*) increased from 19 ± 5 to 36 ± 10 and from 40 ± 6 to 54 ± 10 nmol·min^−1^·100 mL forearm volume^−1^ in PLA and ACI during fasting and clamp conditions, respectively (*P* < 0.05), but was not affected by immobilization. Forearm phenylalanine *R*_d_ was overall higher in ACI than in PLA (*P* = 0.050), but no interactions were observed (all *P* > 0.05). Forearm phenylalanine rate of appearance (*R*_a_; Fig. 5, *G* and *H*) was suppressed during clamps (*P* < 0.05) and was elevated following immobilization (*P* < 0.05). Moreover, forearm phenylalanine *R*_a_ was overall higher in ACI than in PLA (*P* < 0.05), with a tendency for a clamp × treatment interaction (*P* = 0.080). Finally, forearm leucine oxidation (Fig. 5, *I* and *J*) increased from −10 ± 8 in the fasting state to 81 ± 14 nmol·min^−1^·100 mL forearm volume^−1^ during the clamp (*P* < 0.05) and was not affected by immobilization or treatment (*P* > 0.05).

## DISCUSSION

The present study aimed to elucidate the role of positive muscle nonesterified fatty acid (NEFA) balance in immobilization-induced anabolic and insulin resistance by pharmacologically (via oral acipimox administration) suppressing systemic NEFA availability. In contrast to our hypothesis, acipimox administration brought about a greater than twofold elevation of fasting arterialized NEFA concentrations and a greater than fourfold increase in fasting forearm NEFA balance during immobilization. As such, any effect(s) of repeated acipimox administration on circulating NEFA concentrations during 2 days of immobilization had either subsided or were overridden by elevated NEFA availability by the time forearm glucose and amino acid metabolism were determined. Nevertheless, this provided a unique scenario to investigate the role of increased NEFA availability on the anabolic and insulin resistance observed following disuse. Indeed, increased NEFA availability led to an exacerbated decrease in insulin-stimulated forearm glucose uptake in the absence of changes in whole body glucose disposal. Moreover, we demonstrate for the first time that 2 days of forearm immobilization tends to decrease forearm phenylalanine net balance (NB) via an increased rate of phenylalanine appearance (*R*_a_; i.e., release of phenylalanine from muscle to plasma), suggesting increased amino acid efflux from muscle, while muscle amino acid uptake (*R*_d_; i.e., phenylalanine flux from plasma to muscle) was unaffected.

Periods of muscle disuse lead to the substantial development of insulin resistance, i.e., impaired insulin-stimulated glucose uptake, which occurs rapidly following the removal of muscle contraction (10). Here we corroborate previous work (1, 7–9) by demonstrating that immobilization increases forearm NEFA balance ∼2 to 2.5-fold (Fig. 1) and reduced forearm glucose uptake (i.e., direct measure of peripheral muscle insulin sensitivity) by ∼40% under hyperinsulinemic-euglycemic conditions, with hyperaminoacidemic coinfusion (Fig. 3). To understand the interaction between these disuse-induced perturbations of muscle lipid and glucose metabolism, we pharmacologically altered systemic lipid availability via oral acipimox administration. Acipimox is a nicotinic acid analog that can acutely lower plasma NEFA concentrations by 60–75% for a 6-h period (16, 28–31) via the inhibition of adipose tissue lipolysis, with repeated administration over several days previously being reported to improve insulin sensitivity and glucose tolerance in healthy normoglycemic (18, 32, 33) and insulin resistant (16–18) individuals. We assumed that four times daily administration of 250 mg acipimox during 2 days of forearm immobilization would lower plasma NEFA during the entire immobilization period and, at least partially, prevent a positive muscle NEFA balance and subsequent muscle lipid accumulation during disuse. In contrast, however, we observed greater than twofold higher serum NEFA concentrations following immobilization (Fig. 1*B*), during measurements taken ∼10 h after the last acipimox dose. This is in line with a previously reported (29) nocturnal “rebound” effect of acipimox on lipolysis, which has been demonstrated as elevated plasma NEFA concentrations in the morning following repeated acipimox ingestion (34). This nocturnal rebound has been reported as a twofold increase in morning fasting NEFA concentrations following more prolonged acipimox administration (i.e., 2 wk to 3 mo; Refs. 34–36) but not short term (i.e., 2–3 days; Refs. 29, 34). A potential explanation for this observed increase following more chronic supplementation could be an adaptation to maintain long-term energy homeostasis. Indeed, it has been suggested that the rebound rise in NEFA could be a mechanism to compensate for inhibited lipolysis and consequent decreased NEFA concentrations during the night (34). With most work to date conducted almost exclusively in individuals with type 2 diabetes (16, 34–36) using varying dosing protocols and with differences in the timing of the final dose, further work in individuals with normoglycemia is required to elucidate why this rebound effect occurred with its observed magnitude in our study (Fig. 1*B*). Irrespective of the underlying mechanisms, our data suggest that any effect of lowering serum NEFA concentrations *during* the 2 days of immobilization was obfuscated during the measurement period of forearm metabolism following immobilization. Nevertheless, this provided a unique scenario to investigate the effect of an acute increase in NEFA balance on immobilization-induced insulin resistance.

Elevated NEFA availability did not affect whole body glucose disposal (Fig. 2*B*), but participants receiving acipimox demonstrated a greater decrease in insulin-stimulated forearm glucose uptake during forearm immobilization than those supplemented with placebo (Fig. 4, *C*–*F*). Due to tight control of the standardized diet (Table 2) and the lack of group differences observed therein, it is unlikely that the greater peripheral insulin resistance in the acipimox group was caused by dietary intake. We have previously demonstrated that a high-fat, hypercaloric diet (50% excess energy from fat) during 7 days of forearm immobilization did not further exacerbate the positive NEFA balance or muscle insulin resistance induced by disuse (10). Taken together with numerous reports demonstrating that acutely increasing circulating NEFA concentrations causes skeletal muscle insulin resistance and that insulin resistance has plateaued by 24 h of forearm immobilization (11), this would suggest that acutely increasing circulating NEFA causes insulin resistance via a different, albeit transient, mechanism to a lack of contraction per se (e.g., Randle Cycle vs. reduced GLUT4 translocation, respectively; Refs. 13, 37) during disuse. This does not rule out the role of muscle lipid accumulation in disuse-induced insulin resistance (38), but it has important implications for clinical scenarios where circulating lipids are elevated during physical inactivity and food intake requires adequate management, such as during critical illness (39).

To our knowledge, this is the first study to measure muscle amino acid metabolism following merely 2 days of limb immobilization. In line with the rapid (i.e., within 2 days) development of insulin resistance with disuse, the present study also demonstrated a tendency for reduced muscle amino acid net balance (∼2- to 3-fold) under both fasting and clamp conditions during the same timeframe (Fig. 5*A*), which is consistent with our previous observations following one week of disuse (6). Although (despite attempts to methodologically advance imaging techniques) forearm muscle atrophy is not yet measurable via MRI so early into disuse (3), this negative amino acid balance is indicative of early muscle protein loss. Our experimental approach allowed us to estimate that immobilization reduced the forearm net balance of all amino acids during the 30-min clamp steady state from 28.9 to 10.1 mg, representing net uptake of 0.5 and 0.2% of all amino acids infused, respectively. Interestingly, when using the assumptions that 12 h is spent in the fasted state daily, average forearm muscle mass is 0.6 kg (10, 20), and amino acids (as proteins) comprise 84% of human muscle tissue (40), this equates to a theoretical 0.73% daily muscle tissue loss. This is in line with what is observed in short-term leg immobilization studies in which muscle mass was quantified via MRI or CT (2, 41) but is approximately two- to threefold greater than what is typically observed following short-term bed rest (4, 42). This highlights the possibility of measuring early muscle protein loss, predicting subsequent measurable atrophy via imaging methods, directly in forearm muscles in vivo, which can act as an important early target in the development of effective interventional strategies.

Our measurements were conducted under tightly controlled hyperaminoacidemic insulin clamp conditions, which resulted in elevated plasma amino acid concentrations comparable to peak plasma concentrations following ingestion of 35 g whey protein (43). This approach obviated issues associated with applying a hyperinsulinemic-euglycemic clamp only to study “postprandial” amino acid metabolism, whereby circulating amino acid concentrations decrease due to insulin-induced suppression of protein breakdown (44, 45). By combining these clamp conditions with arteriovenous forearm balance measurements and an intravenous stable isotope tracer infusion we were able to demonstrate that the negative muscle protein balance observed after 2 days of immobilization was not due to a reduced phenylalanine *R*_d_, representing muscle amino acid uptake (Fig. 5*E*). This contrasts our previous work in which we demonstrated a small, transient reduction in forearm phenylalanine *R*_d_ following 7 days of forearm immobilization in response to mixed meal ingestion (6). This can potentially be explained by the clamp conditions being more anabolic than mixed meal ingestion, i.e., eliciting higher insulin and amino acid concentrations (Figs. 2*A* and 4*A*, respectively). Although this requires confirmation in further research, this is in line with the potential for supraphysiological insulin concentrations to overcome age-related insulin resistance of protein metabolism (46).

Our data suggest that the removal of contraction per se rapidly induces insulin resistance but does not affect muscle amino acid uptake. This would fit with the different mechanisms and priorities of muscle contraction-mediated glucose and amino acid uptake (i.e., glucose is required as an immediate fuel source), and that the reduced amino acid uptake observed following 7 days of immobilization (6) is a physiological adaptation rather than a reduction in uptake capacity. Interestingly, as the *R*_d_’s measured in the present work are similar to the peak *R*_d_’s measured in response to mixed meal ingestion in our previous work (e.g., ∼50 nmol·min^−1^·100 mL forearm volume^−1^; Ref. 6), this might indicate a maximal uptake capacity for amino acids in forearm muscle tissue. Nonetheless, the negative protein balance within 2 days of immobilization appears to be due to an increase in phenylalanine *R*_a_ with immobilization (Fig. 5*G*). Although it has been suggested that these amino acids may originate from increased muscle protein breakdown (23, 47) this has been debated (48, 49), and we recently showed that 2 days of leg immobilization did not affect fasting and postprandial muscle protein breakdown rates (12). Instead, given amino acid oxidation was not affected by immobilization (Fig. 5*I*; albeit in the face of lower energy demand), it is more likely that impaired muscle protein synthesis, which we have previously shown to occur over 2 days of limb immobilization (12, 23), diverts excess amino acids to the circulation.

The increased NEFA balance and insulin resistance observed with immobilization in the present study is in line with our previous work (6). Specifically, we observed exacerbated immobilization-induced blunting of positive postprandial forearm amino acid balance when NEFA availability was further increased via 7 days of high-fat overfeeding (6). Here we show that four times daily administration of 250 mg acipimox did not affect the immobilization-induced reduction in the net balance of phenylalanine (Fig. 5*B*) and leucine (Fig. 5*D*) nor the phenylalanine *R*_d_ (Fig. 5*F*) or *R*_a_ (Fig. 5*H*), which is in contrast to the negative effect observed on glucose metabolism. Previous studies that have increased lipid availability via dietary means or intravenous infusion approaches have demonstrated reduced whole body protein turnover and muscle amino acid efflux (50–52). In agreement, we have previously demonstrated that acutely elevating NEFA availability combined with a hyperinsulinemic-euglycemic clamp almost completely suppressed the muscle protein synthetic response to feeding (15). This is difficult to reconcile with the present data, particularly given other studies have also demonstrated no effect or even increased muscle protein synthesis with elevated circulating NEFA (53, 54). A possible explanation might be that providing energy from NEFA in the presence of amino acids and insulin creates a more favorable anabolic environment than amino acids and insulin alone but that too much NEFA will lead to muscle lipid accumulation and subsequent impairments in anabolic signaling [e.g., suppressed 4E-BP1 phosphorylation (15)]. This might be particularly relevant in the presence of high insulin (Fig. 2*A*), which will impair NEFA oxidation and release from muscle. Additionally, this will be impacted by the duration of elevation of NEFA concentrations (e.g., acute vs. chronic elevation) and the physiological condition this occurs in [e.g., experimental lipid infusion (15), starvation (55), obesity (56, 57), etc.], which are all factors that affect skeletal muscle amino acid metabolism to different degrees. Importantly, as prolonged disuse (>7 days) leads to intramyocellular lipid accumulation (1, 14), this raises the question of how acipimox administration during prolonged disuse would affect disuse-induced muscle atrophy and metabolic deterioration. Acute acipimox ingestion following short- and longer-term supplementation leads to similar suppression of circulating NEFA concentrations (34). However, based on the greater rebound effect in circulating NEFAs following more prolonged supplementation (discussed above), it can be hypothesized that acipimox supplementation will have diminished potential in attenuating or even preventing lipid-mediated disturbances in muscle amino acid metabolism during more prolonged versus short-term disuse. As such, alternative pharmacological and/or nutritional strategies are warranted to test the effect of lowering lipid availability on disuse-induced anabolic and insulin resistance, and thereby determine the role of perturbed lipid metabolism in the maintenance of muscle mass and metabolic health.

We conclude that rapid muscle insulin resistance observed with 2 days of forearm immobilisation is accompanied by early signs of reduced net muscle amino acid balance in both fasting and insulin-stimulated conditions, which is accompanied by an increase in amino acid efflux from muscle. Acutely elevating circulating NEFA availability with acipimox administration further decreased muscle glucose uptake but did not affect muscle amino acid metabolism. We therefore propose that increased muscle NEFA uptake with removal of muscle contraction may partly contribute to disuse-induced insulin- but not anabolic resistance. The latter thesis requires further investigation given acipimox administration in the present work may have reduced any detrimental effects of muscle lipid accumulation during immobilisation, thereby masking any effects on muscle protein metabolism by a subsequent ‘rebound’ effect of acutely elevated NEFA availability. The effect of lowering circulating NEFA on muscle deterioration during disuse remains to be investigated.

## DATA AVAILABILITY

Data will be made available upon reasonable request.

## GRANTS

This research was funded in whole by the Wellcome Trust 209198/Z/17/Z. A.J.M. and D.R.A. are supported in part by National Institute of Aging Grant P30-AG024832.

## DISCLOSURES

None of the authors disclose any conflicts of interest.

## AUTHOR CONTRIBUTIONS

M.L.D. and F.B.S. conceived and designed research; M.L.D., T.S.O.J., and M.V.D. performed experiments; M.L.D., T.S.O.J., D.R.A., and A.J.M. analyzed data; M.L.D., R.C.A., B.T.W., and F.B.S. interpreted results of experiments; M.L.D. prepared figures; M.L.D. drafted manuscript; M.L.D., R.C.A., B.T.W., and F.B.S. edited and revised manuscript; M.L.D., T.S.O.J., R.C.A., M.V.D., D.R.A., A.J.M., B.T.W., and F.B.S. approved final version of manuscript.
